# Inferior Pole Scaphoid Nonunion in a 12-Year-Old Boy: Lessons on Compliance, Follow-Up, and Surgical Salvage—A Case Report

**DOI:** 10.3390/reports9030252

**Published:** 2026-08-03

**Authors:** Adnan Hussain Alrashed, Abdullah Abdulhadi Alamer, Mohammed Jassim Alhassan, Abdullah Mansour Alkhars, Fatimah Mustafa Althabit, Mashael Abdulrahman Alhussain, Abdullah Fahmi Alkhars

**Affiliations:** 1 King Fahad Hospital Hufof, Al Ahsa 13881, Saudi Arabia; 2 Almanaa Specialist Hospital, Al Ahsa 13881, Saudi Arabia; 3 King Faisal General Hospital, Al Ahsa 13881, Saudi Arabia; 4 College of Medicine, King Faisal University, Al Ahsa 13881, Saudi Arabia; 5 King Abdulaziz Hospital of the National Guard, Al Ahsa 13881, Saudi Arabia

**Keywords:** scaphoid nonunion, pediatric, inferior pole, iliac-crest autograft, headless compression screw

## Abstract

**Background and Clinical Significance:** Scaphoid fractures and nonunion are uncommon in skeletally immature patients. Pediatric nonunion most often follows a missed or delayed diagnosis or failure of conservative treatment. Inferior-pole nonunion is particularly uncommon, and evidence guiding graft selection in children is limited. We report a case in which preoperative and intraoperative assessment of fragment viability supported the use of a non-vascularized graft. **Case presentation:** A 12-year-old boy sustained a right inferior-pole scaphoid fracture after falling onto an outstretched hand. The fracture was missed at the initial emergency-department visit. Thumb-spica immobilization was subsequently prescribed, but the patient repeatedly removed the cast, missed appointments, and was lost to follow-up. At referral six months after injury, radiographs and multiplanar CT demonstrated established inferior-pole nonunion. MRI showed preserved marrow fat signal in both fragments without osteonecrosis. Open reduction and internal fixation were performed through a dorsal approach using a 2.4 mm headless compression screw and approximately 1 cc of cancellous iliac-crest autograft. Intraoperatively, both fragments appeared viable, without cystic or sclerotic change. At two months, the patient was pain-free and radiographs showed progressing union. The Quick Disabilities of the Arm, Shoulder and Hand (QuickDASH) score improved from 25 preoperatively to 10 at two months. CT at six months confirmed complete osseous union, with a QuickDASH score of 0. At 1.5 years, he remained pain-free, had full flexion with a 5° terminal extension lag, and had returned to table tennis without functional limitation. **Conclusions:** In this inferior-pole scaphoid nonunion, open reduction and internal fixation with iliac-crest cancellous autograft achieved CT-confirmed union and sustained functional recovery. MRI and intraoperative confirmation of viable bone supported selection of a non-vascularized graft. At 1.5 years, the patient was pain-free, had returned to sport without functional limitation, and had a QuickDASH score of 0.

## 1. Introduction

Fractures of the scaphoid are uncommon in skeletally immature patients, and nonunion is even more uncommon. The partly cartilaginous structure, strong periosteum and remodeling ability of the bone generally make healing more likely and pediatric nonunion is more likely to be caused by missed or late diagnosis or failure of conservative treatment than intrinsic failure of the bone to heal [1,2,3]. Scaphoid is a bridge between the proximal and distal carpal rows and is responsible for load bearing across the wrist. Impaired healing is most likely to occur in the waist and proximal pole where the blood supply is mostly retrograde [4,5]. Reported pediatric scaphoid nonunions therefore are primarily at these locations, and distal or inferior-pole nonunion is rare and has only been reported in small series and isolated cases [1,6].

The viability of fragments is key to management as they will affect the choice of graft: if the fragments are viable, a non-vascularized cancellous graft can be considered, but with compromised vascularity a vascularized graft may be indicated. The open distal radial physis also influences donor-site selection in children and has led to the use of alternative sources of grafts [7,8,9,10].

Previously, the treatment of distal scaphoid nonunion in a 12-year-old patient has been described using arthroscopic debridement, bone substitute and K-wire fixation [6]. The present report describes the case of an inferior-pole nonunion that was treated with open fixation using a dorsal headless screw and with autologous cancellous grafting from the iliac crest following MRI and intraoperative assessment of fragment viability. It is not just rare, but also has educational value in its explicit biology-based rationale for graft selection, stable screw fixation, CT-confirmed union, and longer-term functional follow-up. The report is based on the CARE guidelines (Table S1 in the Supplementary Materials).

## 2. Case Presentation

### 2.1. Patient Information

**Figure 1:**
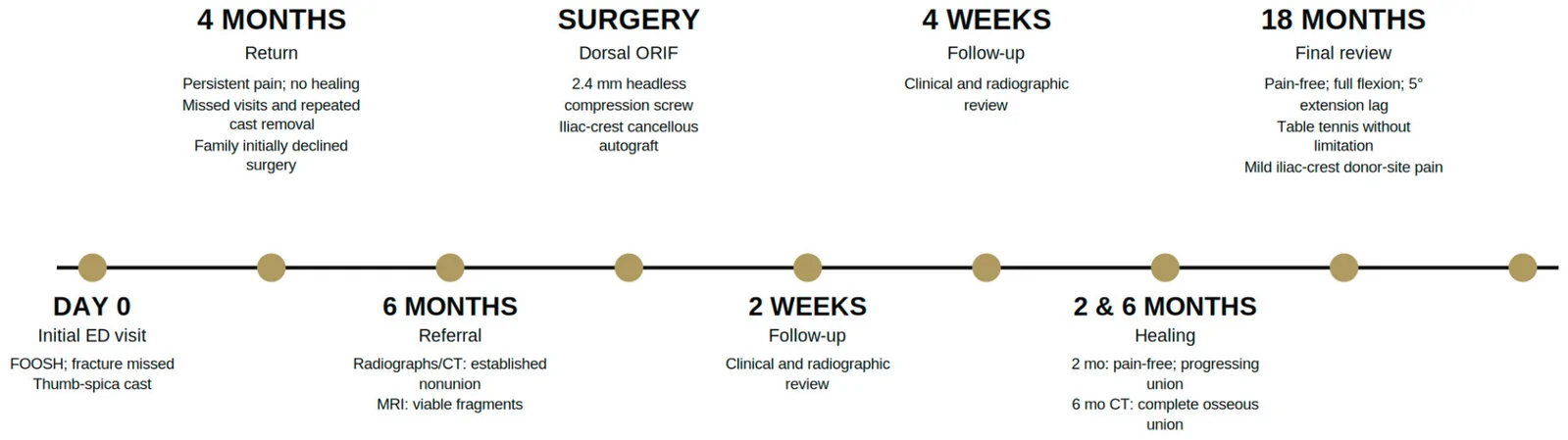
Clinical timeline of the patient’s presentation.

A 12-year-old right-handed boy fell on an outstretched right hand, resulting in an isolated right-wrist injury. His medical, developmental, and family history were otherwise unremarkable, and he had no previous wrist injury or medication. The timeline of the patient’s presentation is depicted in Figure 1.

### 2.2. Clinical Findings

He was alert and oriented (GCS 15/15) at presentation. Physical exam revealed only mild swelling of the wrist, tenderness over the anatomic snuffbox with no deformity and intact distal neurovascular examination.

### 2.3. Diagnostic Assessment

**Figure 2:**
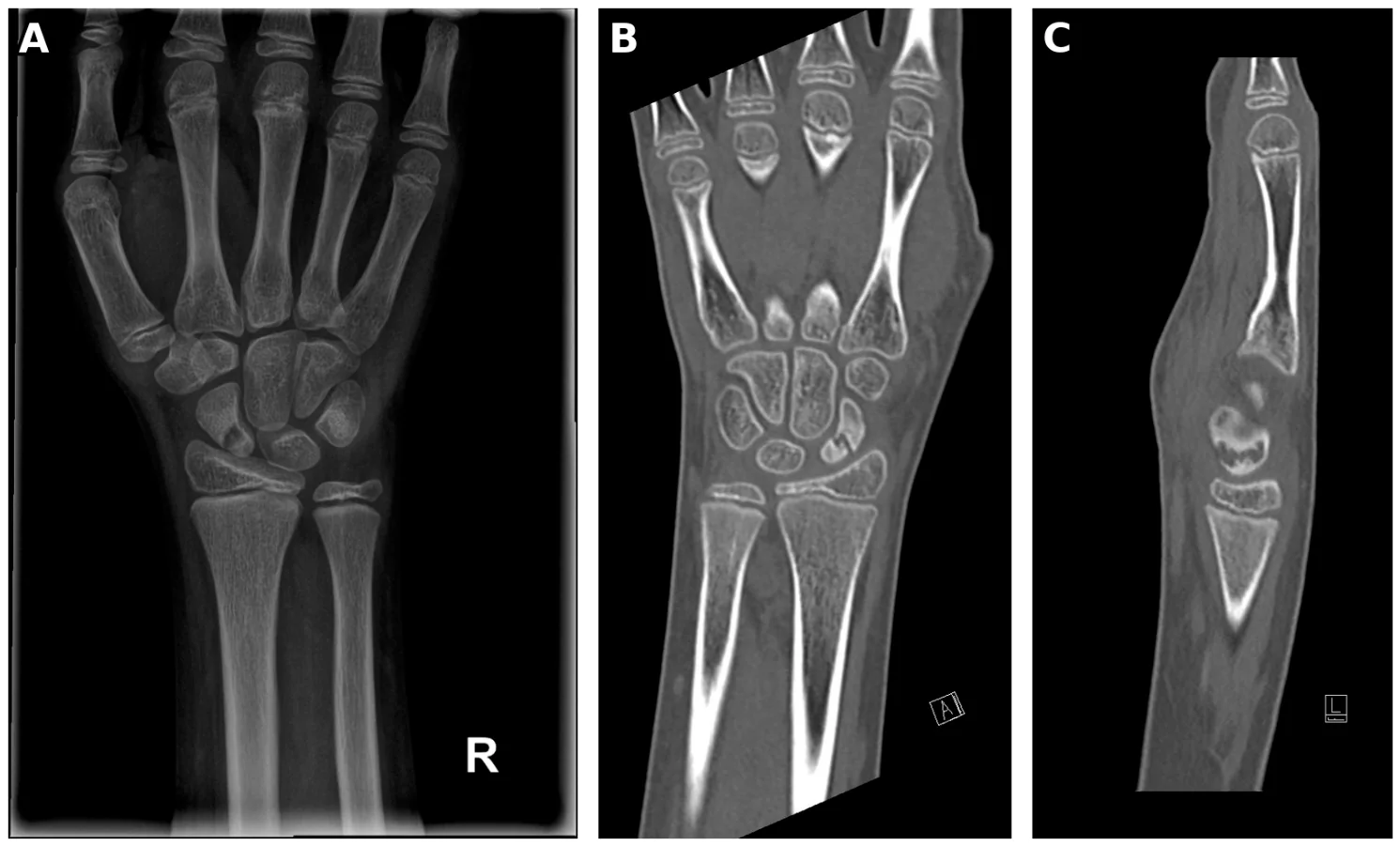
Diagnostic imaging of the inferior-pole scaphoid nonunion. (**A**) Initial posteroanterior radiograph retrospectively showing a nondisplaced or minimally displaced inferior-pole fracture that was missed at the initial emergency-department visit. (**B**) Coronal and (**C**) sagittal CT reconstructions obtained at referral, demonstrating a persistent fracture line, corticated margins, interfragmentary separation, and absence of bridging trabeculae.

At the initial emergency-department visit, the inferior-pole scaphoid fracture was not recognized. Retrospective review of the initial posteroanterior and lateral X-rays revealed a nondisplaced or minimally displaced inferior-pole fracture (Figure 2A, lateral view Supplementary Figure S1). Reduction was not done since there was no clinically significant displacement.

A thumb-spica cast was then applied for immobilization of the wrist. At follow-up, however, the patient repeatedly pulled off the cast and failed to attend several follow-up appointments. Ultimately, he took out the final cast himself, and was lost to follow-up. On return four months post-injury, he complained of ongoing wrist pain. On examination there was scaphoid region tenderness but full wrist range of motion and serial radiographs demonstrated persistence of the fracture without signs of healing. The family refused to have surgery. Referral radiographs and coronal and sagittal CT reconstructions 2 months later revealed a nonunion at the inferior pole with a persistent fracture line, corticated and sclerotic margins, interfragmentary separation, and lack of bridging trabeculae (Figure 2B,C; Supplementary Figures S2–S4). Persistent clinical and imaging findings 6 months after injury were used to diagnose nonunion.

**Figure 3:**
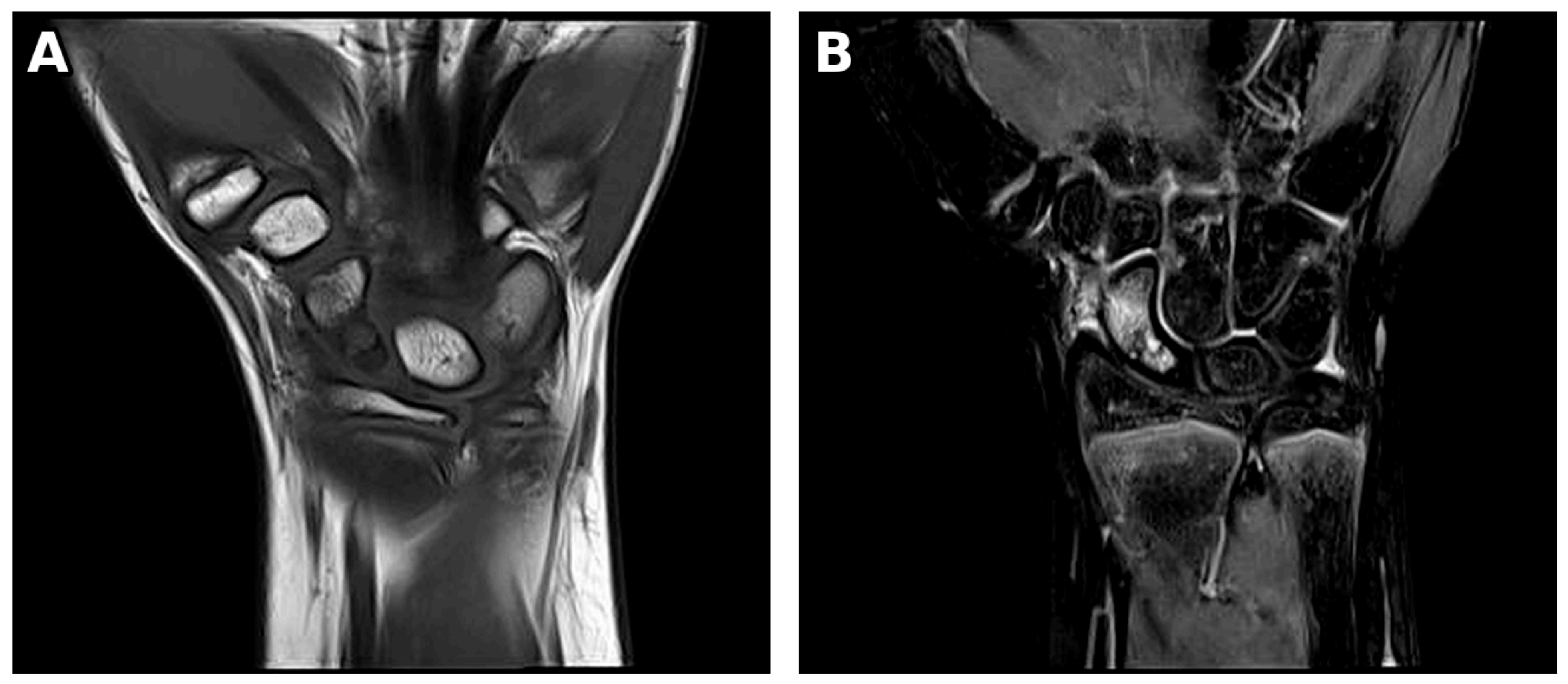
MRI assessment of scaphoid viability. (**A**) Coronal T1-weighted image showing preserved fatty marrow signal in both fragments. (**B**) Coronal fat-suppressed fluid-sensitive image showing increased signal at the nonunion interface. No MRI evidence of osteonecrosis was identified.

Preop MRI was done to determine fragment viability. Available coronal T1-weighted images revealed preserved fatty marrow signal in both scaphoid fragments, and a coronal fat saturated, fluid-sensitive image revealed increased signal at the nonunion interface (Figure 3; Supplementary Figure S6). Preservation of fragment viability was noted by the lack of MRI evidence of osteonecrosis.

### 2.4. Therapeutic Intervention

The nonunion was exposed and inspected via a dorsal approach. No cystic change or sclerosis was noted and the bone was considered to be healthy and viable. The fracture site was prepared and reduced and fixation was done with a 2.4 mm headless compression screw placed through the proximal pole. A second 2–3 cm incision was made in the iliac crest for cancellous autograft. The external oblique was lifted up to lay bare the iliac crest and the iliac apophysis was split and a window of the cortex created with an osteotome. A total of about 1 cc of graft was removed from the supra-acetabular region and was placed in the nonunion site.

### 2.5. Follow-Up and Outcomes

**Figure 4:**
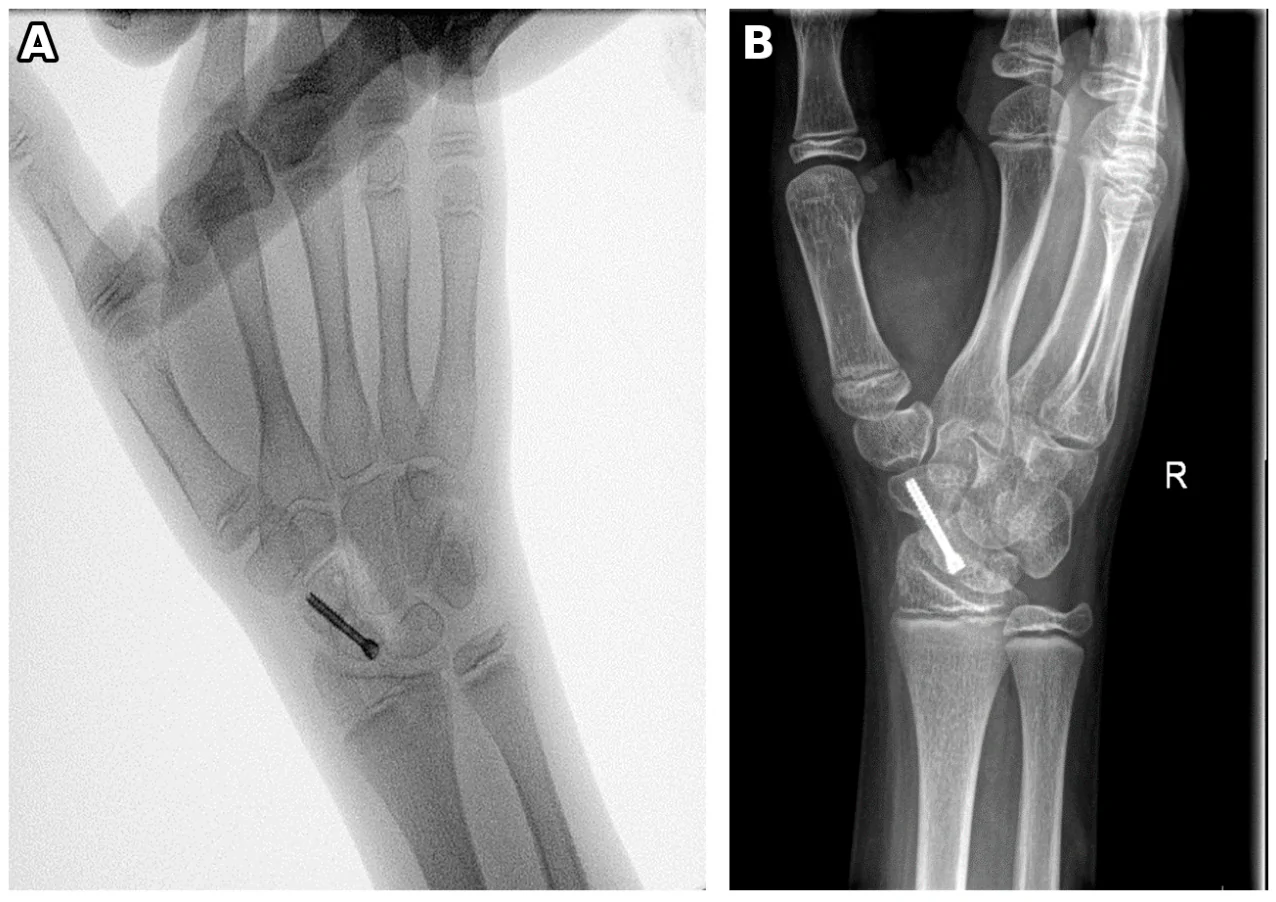
Fixation and early healing. (**A**) Immediate postoperative posteroanterior radiograph demonstrating the 2.4 mm headless compression screw and restored scaphoid alignment. (**B**) Posteroanterior radiograph obtained at two months demonstrating progressing union. Corresponding lateral views are provided in Supplementary Figure S5.

**Figure 5:**
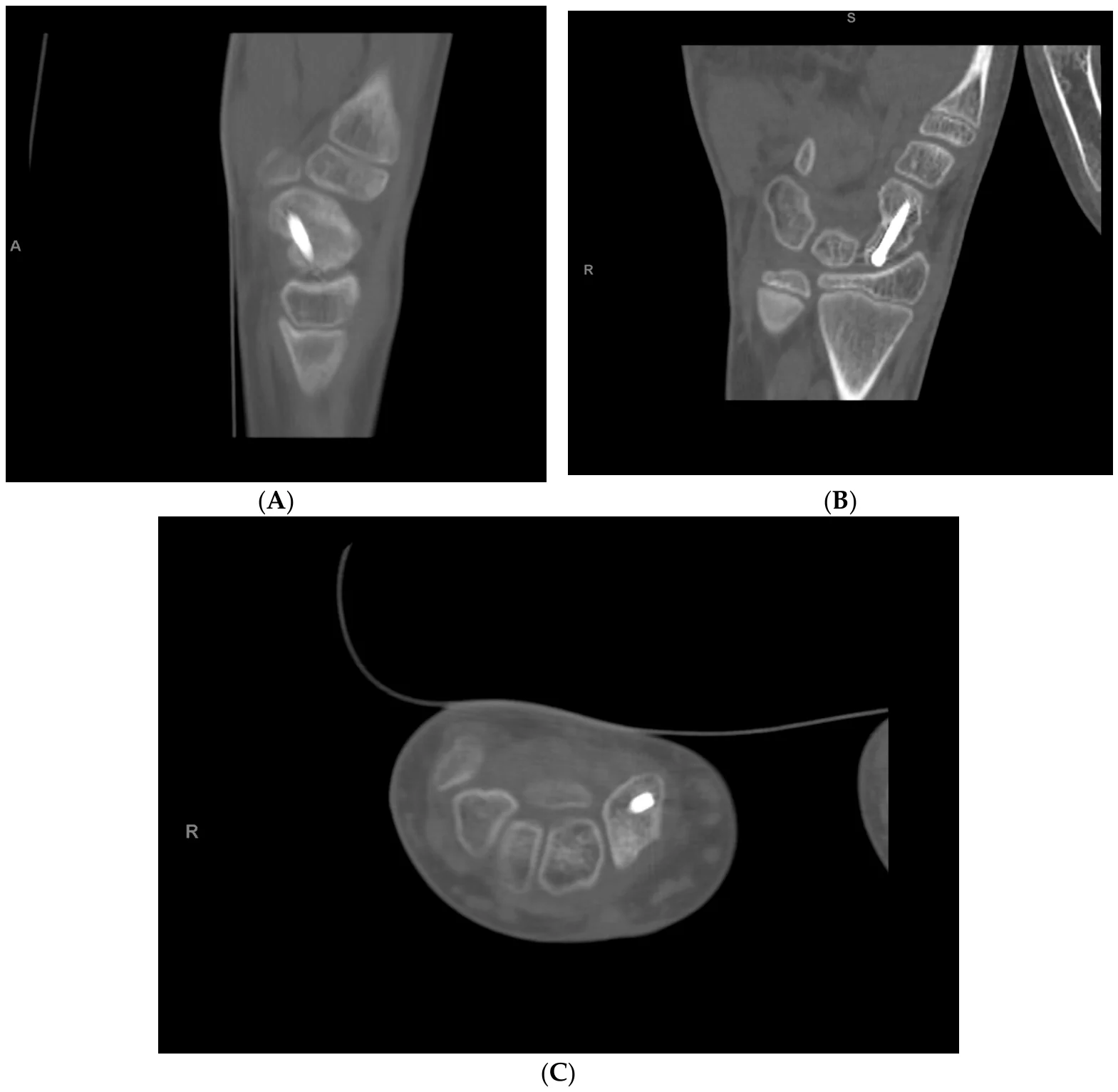
Six-month CT confirmation of union. (**A**) Sagittal, (**B**) coronal, and (**C**) axial CT images demonstrating bridging trabeculae across the former inferior-pole nonunion, confirming complete osseous union with maintained scaphoid alignment and stable screw position.

Postoperative follow-up was performed at 2 weeks, 4 weeks, 2 months and 6 months. Post-op X-rays showed good screw position and scaphoid alignment (Figure 4A). The wrist was pain-free at two months and X-rays demonstrated progressive union (Figure 4B). The Quick Disabilities of the Arm, Shoulder and Hand (QuickDASH) score preoperatively was 45 and at two months was 10. At six months, multiplanar CT demonstrated bridging trabeculae across the former nonunion and confirmed complete osseous union (Figure 5); the QuickDASH score was 0.

During review at the end of the 1.5 years, the patient reported no wrist pain or functional limitations and was completely pain-free. He had a QuickDASH score of 0. He had complete wrist flexion with 5° terminal lag that further improved with physiotherapy. He is now playing table tennis freely, and is very happy with the outcome. His only remaining complaint was mild pain at the iliac-crest donor site. Formal grip strength, numerical flexion measurements, PRWE and other standardized patient-reported outcome measures were not obtained.

### 2.6. Patient Perspective

The patient and his guardians gave high level of satisfaction with the intervention. No functional limitations were reported, and he was free of pain, except for mild pain at the iliac-crest donor site, and was able to play table tennis without pain.

## 3. Discussion

This case is novel, not only because of the rarity of pediatric scaphoid nonunion. In a few reports, Oestreich et al. reported one distal-pole nonunion in an 18-patient series [1], and Lee and Kim reported one distal nonunion that was treated arthroscopically with bone substitute and K-wires in 12-year-old [6]. The present inferior-pole nonunion, however, was treated in a clearly biology-driven manner: MRI and direct inspection at surgery showed that the fragments were viable—this made non-vascularized autologous grafting the treatment of choice—a dorsal headless compression screw was used to provide stable fixation—and complete union was confirmed by CT scans—with functional recovery at 1.5 years.

Several modifiable care-pathway factors can also converge, as in this case. Initial fracture nonrecognition missed appointments and loss to follow-up interrupted immobilization and surveillance, and family initially refused surgery when healing failed. These events highlight the importance of careful assessment of pediatric scaphoid radiographs, clear counseling on cast compliance and structured follow-up with early re-evaluation if symptoms do not resolve.

The patient remained symptomatic and CT showed nonunion 6 months after injury with corticated and sclerotic margins, interfragmentary separation, and no bridging trabeculae, which indicated surgery. Open treatment permitted direct debridement, reduction and confirmation of punctate viable bone. The preserved marrow fat signal on T1-weighted MRI and the healthy appearance of the intraoperative specimen led to the decision to perform a non-vascularized cancellous graft rather than a vascularized graft. Iliac crest served as an osteogenic graft, but did not compromise the open distal radial physis [1,6,7,8,10].

The dorsal compression screw with headless compression was chosen to allow interfragmentary compression and stable fixation of the nonunion across the small inferior pole and to allow direct evaluation of the nonunion through the open approach. This is a different case from the 12-year-old distal-pole case previously reported treated with arthroscopic debridement, bone substitute and K-wires [6]. Table 1 has been modified to distinguish union timing and confirmation method, final follow-up, functional outcomes and complications, and compares the present outcome with selected pediatric and adolescent reports.

At 6 months, CT showed complete osseous union and at 1.5 years the patient was pain-free with full flexion and only a 5° terminal extension lag and was able to play table tennis without restriction. The QuickDASH score was 25 preoperatively, 10 at 2 months, 0 at 6 months, and 0 at 1.5 years. There was a reported mild pain at the iliac-crest donor site, which is a known disadvantage of this donor site. The lack of formal grip-strength testing, numerical flexion measurements and another wrist-specific patient-reported outcome like PRWE are limitations of interpretation. However, the case suggests that graft biology and fixation strategy should be matched to the viability, fracture configuration and skeletal maturity of the fragment, and a reliable follow-up should be maintained to document union and function.

## 4. Conclusions

Open reduction and fixation with iliac-crest cancellous autograft achieved CT-confirmed union in this child with inferior-pole scaphoid nonunion. MRI and intraoperative findings confirmed fragment viability and were used to support the use of a non-vascularized graft. At 1.5 years the patient was pain-free and able to resume table tennis without functional limitation, and had only a 5° terminal extension lag, mild iliac-crest donor-site pain, and a QuickDASH score of 0.

## Figures and Tables

**Table 1:** Selected published pediatric and adolescent scaphoid-nonunion cases, including union confirmation, follow-up, complications, and functional outcomes. The present case is highlighted.

Study	N; Age/Sex	Pattern; Delay	Graft and Fixation	Union: Timing and Confirmation	Final Follow-Up and Functional Outcome	Complications
Present case	1; 12/M	Inferior-pole nonunion; missed fracture; 6 mo	Iliac cancellous autograft; dorsal ORIF; 2.4 mm headless compression screw	Complete union at 6 mo; multiplanar CT	18 mo; QuickDASH 0; pain-free; full flexion; 5° terminal extension lag; table tennis without limitation	Mild iliac-crest donor-site pain
Lee & Kim 2021, case 1 [6]	1; 15/M	Waist nonunion with humpback; ~12 mo	Iliac cancellous autograft; arthroscopy-assisted grafting; K-wires	Union at 9 wk on serial imaging; CT shown at final FU	54 mo; VAS 3→0; QuickDASH 29.5→0	None reported
Lee & Kim 2021, case 2 [6]	1; 15/M	Waist nonunion; 18 mo	Iliac cancellous autograft; arthroscopy-assisted grafting; K-wires	Union at 10 wk on serial imaging; CT shown at final FU	Final FU reported; pain and ROM improved	None reported
Lee & Kim 2021, case 3 [6]	1; 12/M	Distal-pole nonunion; 3 mo	Bone substitute; arthroscopic debridement; K-wires	Union at 3 mo; serial radiographs/CT	34 mo; no pain; ROM ~96%; grip ~69%; QuickDASH 63.8→6.8	None reported
Bandinelli et al. 2025 [7]	3; 13–17/M	Complex waist nonunion; 5–18 mo; one failed surgery	Volar distal-radius VBG; K-wires	Union at ~3 mo; serial radiographs	Clinical FU 3 yr; final radiographs ~18 mo; DASH ~5–13% at 1 yr	No growth-plate complication; secondary wire removal
Oestreich et al. 2020 [1]	18; 9–15/15M, 3F	Waist 11; proximal 6; distal 1; mean delay ~8 mo	Immobilization or ORIF ± graft; techniques varied	Healing reported after operative or nonoperative care; radiographs with selective CT/MRI	Study-specific FU varied; excellent outcomes with few complications	Few complications reported
Lin et al. 2022 [11]	10; mean 14.3 yr/9M, 1F	Waist 8; proximal 2; mean 33 wk to evaluation	Distal-radius cancellous autograft; screw (n = 9) or screw + K-wire (n = 1)	9/10 after index surgery; mean 17 ± 5 wk; radiographs; revision achieved union in 1	All returned fully to activity; final FU duration NR in abstract	One persistent nonunion requiring revision
Koshio et al. 2026 [10]	4; ≤14 yr	Nonunion with open epiphysis; delay NR	Second-metacarpal-base VBG; headless screw + K-wires	Union in all; confirmation method/timing NR in abstract	Favorable ROM and Mayo Wrist Scores; FU duration NR in abstract	One incomplete DISI correction

*Data are presented as reported in the original studies. CT, computed tomography; DASH, Disabilities of the Arm, Shoulder and Hand questionnaire; DISI, dorsal intercalated segment instability; F, female; FU, follow-up; K-wires, Kirschner wires; M, male; NR, not reported; ORIF, open reduction and internal fixation; PRWE, Patient-Rated Wrist Evaluation; QuickDASH, abbreviated Disabilities of the Arm, Shoulder and Hand questionnaire; ROM, range of motion; VAS, visual analog scale; VBG, vascularized bone graft. The symbol ~ indicates an approximate value.*

## Data Availability

The data presented in this case report are available within the article. Additional data are not publicly available due to patient privacy and confidentiality considerations.

## Supplementary Materials

The following supporting information can be downloaded at: https://www.mdpi.com/article/10.3390/reports9030252/s1, Figure S1: Initial lateral radiograph of the right wrist, retrospectively showing the inferior-pole scaphoid fracture that was missed at the initial emergency-department visit (companion to Figure 1A); Figure S2: Posteroanterior and lateral radiographs obtained during conservative follow-up, demonstrating established inferior-pole scaphoid nonunion; Figure S3: Subsequent preoperative radiographs showing persistent inferior-pole scaphoid nonunion. (A) Posteroanterior and (B) lateral views; Figure S4: Additional CT reconstructions obtained at referral. (A) Coronal and (B) oblique coronal views demonstrating the inferior-pole nonunion. Coronal and sagittal images in the main manuscript provide orthogonal assessment; a 3D reconstruction was not available; Figure S5: Lateral radiographs of fixation and early healing (companions to Figure 3). (A) Immediate postoperative and (B) two-month lateral views; Figure S6: Additional preoperative coronal T1-weighted MRI image of the wrist (companion to Figure 2), showing preserved marrow fat signal in the scaphoid fragments. Table S1: CARE Checklist (2013).
