# Supplementary material for: Inferior Pole Scaphoid Nonunion in a 12-Year-Old Boy: Lessons on Compliance, Follow-Up, and Surgical Salvage—A Case Report
Source: Reports (MDPI). 2026 Aug 3;9(3):252. doi: 10.3390/reports9030252 (PMC13510641; doi:10.3390/reports9030252)
Supplement: Supplementary file 1 [file reports-09-00252-s001.zip › reports-4427845-supplementary.pdf]

Supplementary Materials

Inferior Pole Scaphoid Nonunion in a 12-Year-Old Boy: Lessons on Compliance, Follow-Up, and Surgical Salvage—A Case Report

Supplementary Figures

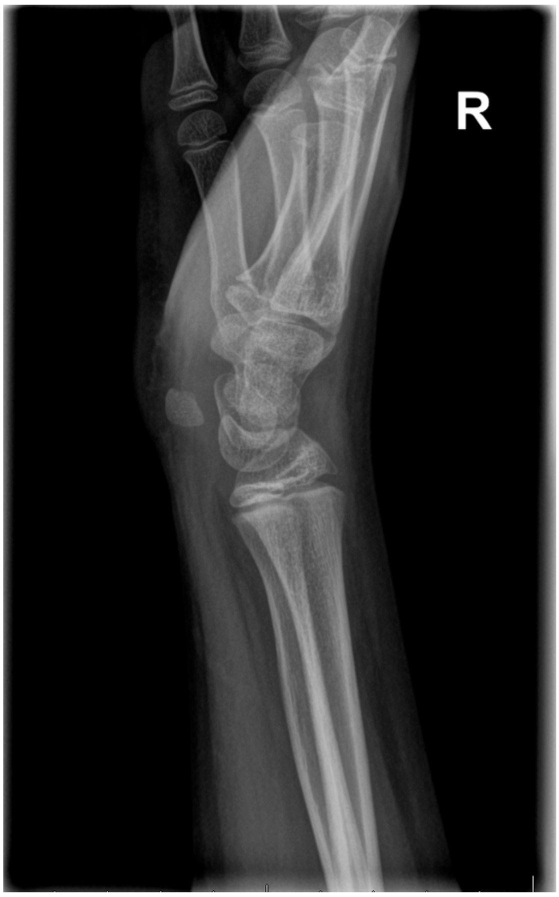

Figure S1. Initial lateral radiograph of the right wrist, retrospectively showing the inferior-pole scaphoid fracture that was missed at the initial emergency-department visit (companion to Figure 1A).

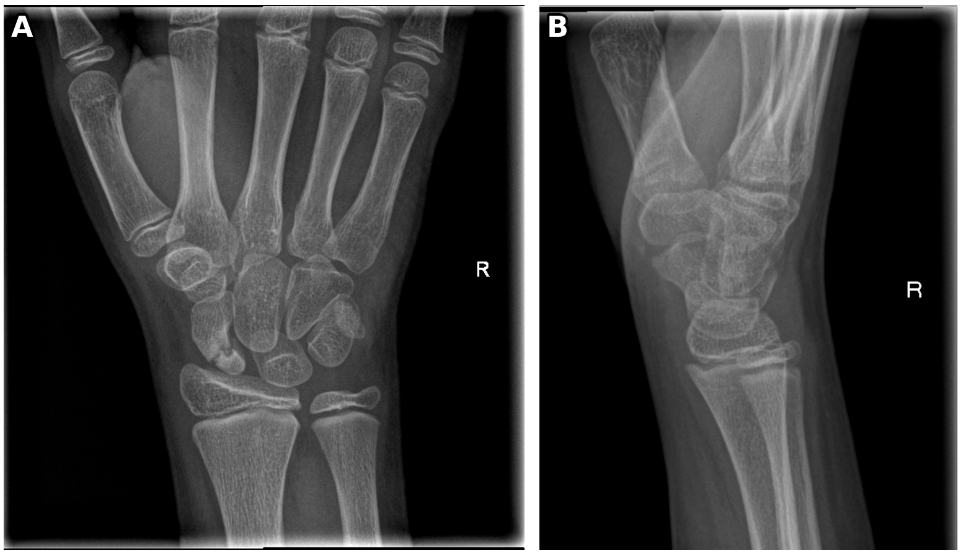

Figure S2. Posteroanterior and lateral radiographs obtained during conservative follow-up, demonstrating established inferior-pole scaphoid nonunion.

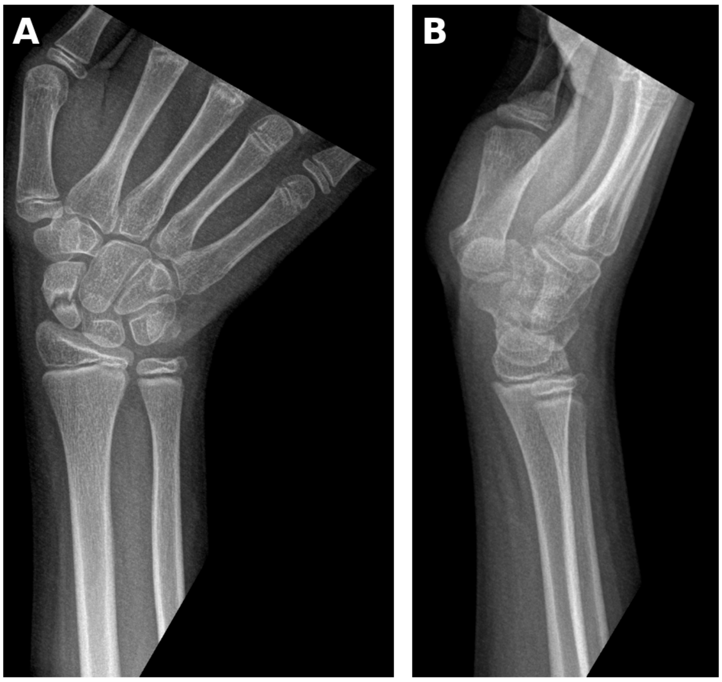

Figure S3. Subsequent preoperative radiographs showing persistent inferior-pole scaphoid nonunion. (A) Posteroanterior and (B) lateral views.

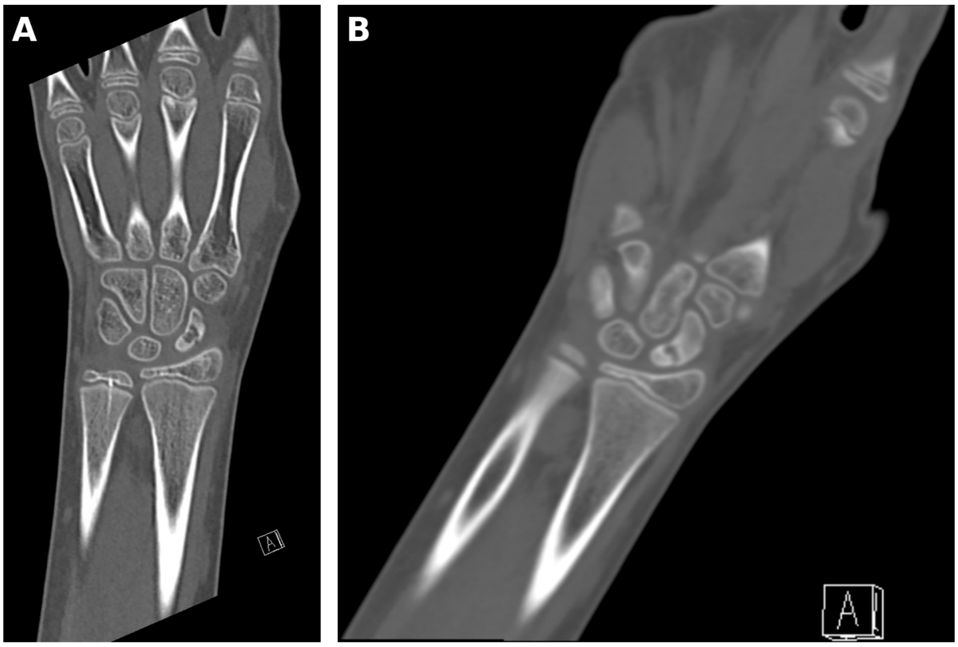

Figure S4. Additional CT reconstructions obtained at referral. (A) Coronal and (B) oblique coronal views demonstrating the inferior-pole nonunion. Coronal and sagittal images in the main manuscript provide orthogonal assessment; a 3D reconstruction was not available.

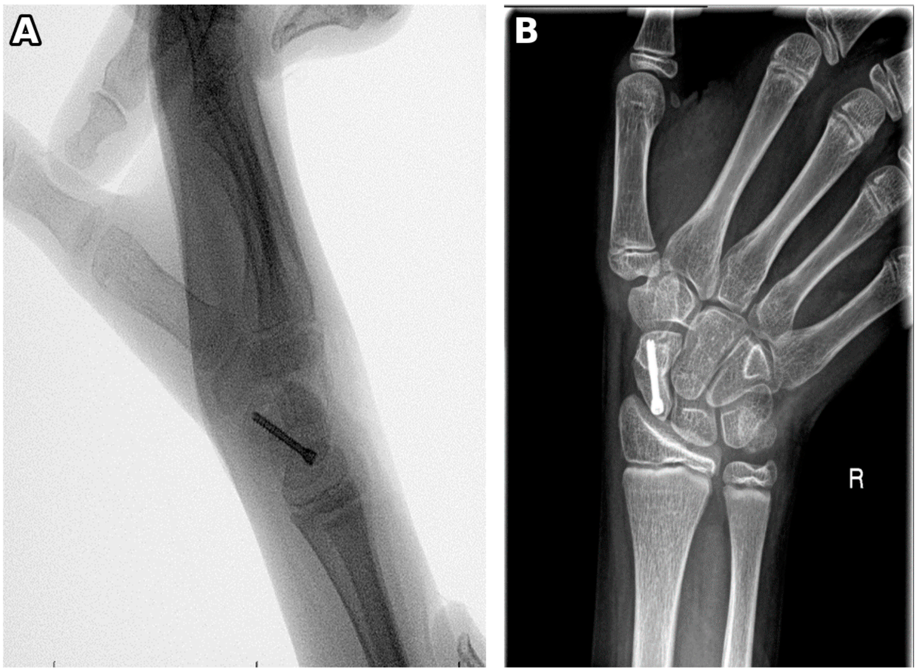

Figure S5. Lateral radiographs of fixation and early healing (companions to Figure 3). (A) Immediate postoperative and (B) two-month lateral views.

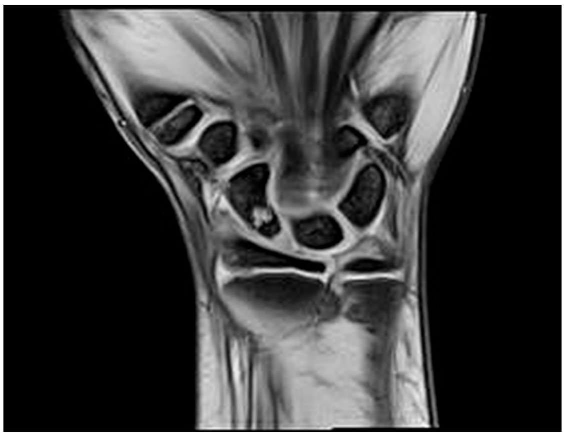

Figure S6. Additional preoperative coronal T1-weighted MRI image of the wrist (companion to Figure 2), showing preserved marrow fat signal in the scaphoid fragments.

Table S1. CARE Checklist (2013)

The checklist below identifies the location of each CARE item and transparently notes information that was not recorded or was unavailable.

#	CARE item	Location / status in revised manuscript
1	Title — "case report" in the title	Title (case report identified)
2	Key words (2–5)	Keywords (5 terms)
3	Abstract — background, case summary, conclusion	Structured Abstract
4	Introduction — background and rationale	Introduction (paediatric focus and comparative novelty)
5a	Patient information — demographics, history	Patient Information
5b	Clinical findings	Clinical Findings
6	Timeline	Chronological narrative in Case Presentation; calendar dates intentionally omitted because unavailable
7	Diagnostic assessment — methods, challenges, diagnosis, prognosis	Diagnostic Assessment; Figures 1-2; imaging criteria for nonunion stated
8	Therapeutic intervention — types and administration	Therapeutic Intervention; operative technique detailed; postoperative protocol unavailable and acknowledged as a limitation
9	Follow-up and outcomes — clinician & patient-assessed	Follow-up and Outcomes; Figure 3; return to sport, extension deficit, and donor-site symptoms reported; formal metrics unavailable
10	Discussion — strengths, limitations, literature, rationale	Discussion (case-focused rationale, comparison, strengths, and limitations)

#	CARE item	Location / status in revised manuscript
11	Patient perspective	Patient Perspective section; formal patient-authored perspective not obtained
12	Informed consent	Informed Consent Statement (legal guardian)
